# Radiogenomic association of deep MR imaging features with genomic profiles and clinical characteristics in breast cancer

**DOI:** 10.1186/s40364-023-00455-y

**Published:** 2023-01-24

**Authors:** Qian Liu, Pingzhao Hu

**Affiliations:** 1 Department of Biochemistry and Medical Genetics, University of Manitoba, 745 Bannatyne Avenue, Winnipeg, MB R3E 0J9 Canada; 2 Department of Computer Science, University of Manitoba, E2-445 EITC, Winnipeg, MB R3T 2N2 Canada; 3 Department of Statistics, University of Manitoba, 318 Machray Hall, Winnipeg, MB R3T 2N2 Canada; 4 CancerCare Manitoba Research Institute, 675 McDermot Avenue, Winnipeg, MB R3E 0V9 Canada; 5 Department of Biochemistry, Western University, Medical Sciences Building Rm. 342, London, ON N6A 5C1 Canada

**Keywords:** Radiogenomics, Deep learning, Breast cancer, Medical imaging, Denoise autoencoder

## Abstract

**Background:**

It has been believed that traditional handcrafted radiomic features extracted from magnetic resonance imaging (MRI) of tumors are normally shallow and low-ordered. Recent advancement in deep learning technology shows that the high-order deep radiomic features extracted automatically from tumor images can capture tumor heterogeneity in a more efficient way. We hypothesize that MRI-based deep radiomic phenotypes have significant associations with molecular profiles of breast cancer tumors. We aim to identify deep radiomic features (DRFs) from MRI, evaluate their significance in predicting breast cancer (BC) clinical characteristics and explore their associations with multi-level genomic factors.

**Methods:**

A denoising autoencoder was built to retrospectively extract 4,096 DRFs from 110 BC patients’ MRI. Visualization and clustering were applied to these DRFs. Linear Mixed Effect models were used to test their associations with multi-level genomic features (GFs) (risk genes, gene signatures, and biological pathway activities) extracted from the same patients’ mRNA expression profile. A Least Absolute Shrinkage and Selection Operator model was used to identify the most predictive DRFs for each clinical characteristic (tumor size (T), lymph node metastasis (N), estrogen receptor (ER), progesterone receptor (PR), and human epidermal growth factor receptor 2 (HER2) status).

**Results:**

Thirty-six conventional radiomic features (CRFs) for 87 of the 110 BC patients provided by a previous study were used for comparison. More than 1,000 DRFs were associated with the risk genes, gene signatures, and biological pathways activities (adjusted *P*-value < 0.05). DRFs produced better performance in predicting T, N, ER, PR, and HER2 status (AUC > 0.9) using DRFs. These DRFs showed significant powers of stratifying patients, linking to relevant biological and clinical characteristics. As a contrast, only eight risk genes were associated with CRFs. The RFs performed worse in predicting clinical characteristics than DRFs.

**Conclusions:**

The deep learning-based auto MRI features perform better in predicting BC clinical characteristics, which are more significantly associated with GFs than traditional semi-auto MRI features. Our radiogenomic approach for identifying MRI-based imaging signatures may pave potential pathways for the discovery of genetic mechanisms regulating specific tumor phenotypes and may enable a more rapid innovation of novel imaging modalities, hence accelerating their translation to personalized medicine.

**Supplementary Information:**

The online version contains supplementary material available at 10.1186/s40364-023-00455-y.

## Background

Breast cancer (BC) is the most commonly diagnosed cancer and the second leading cause of cancer death for women [1]. BC is a polygenetic disease, and the risk of developing it is influenced by multiple genes. Many efforts in genomics were made to identify BC-associated biomarkers so that better clinical decisions could be made. Genomics has improved today’s medicine tremendously, but techniques like the next generation sequencing (NGS) used in genomic experiments are costly, invasive, and only representing the information of a small tumor tissue bulk. Magnetic resonance imaging (MRI) is widely involved in disease management [2] due to its non-invasive and ability to view the ﻿entire tumor and surrounding parenchyma [3]. However, traditional human experience-based imaging diagnosis is criticized for its subjectivity. Therefore, radiomics was developed to extract high-throughput image features using advanced mathematical algorithms.

The integration of radiomics with genomics led to the radiogenomics. Burnside et al. reported 36 conventional radiomic features (CRFs) extracted from BC patients’ MRI [4]. These CRFs were further evaluated as having genomic significance [5, 6]. However, since these CRFs were also obtained under radiologists’ prior knowledge, their objectivity was still doubted. In fact, most radiogenomic studies were done in a semiautomatic way. There is a pressing need to explore fully automatic algorithms in radiogenomics.

Deep learning (DL) is very successful in solving computer vision problems. Recently, DL has been introduced to radiomics. Li et al. developed a DL model to automatically extract deep radiomic features (DRFs) from glioma MRI in a supervised way [7]. However, in exploratory research, it is more reasonable to extract DRFs in an unsupervised way. Because features extracted in a data-driven manner using unsupervised approaches have higher flexibility of representing data intrinsic patterns than supervised hypothesis-driven methods [3]. It is likely that a supervised model will force the features to just represent the label information used to train the model, instead of representing data intrinsic patterns. Li et al. further proved that their DRFs were associated with glioma tumor grading [7]. However, they didn’t perform further genomic exploration of their DRFs.

DL-based radiogenomics was unexplored, especially in current BC studies. In this work, we hypothesize that unsupervised DL-based auto- MRI DRFs have significant associations with genomic profiles of BC, and these DRFs could predict patients’ clinical characteristics. We also hypothesize that the visualized DRFs could be explained semantically.

## Material and methods

### Data sources

**Fig. 1:**
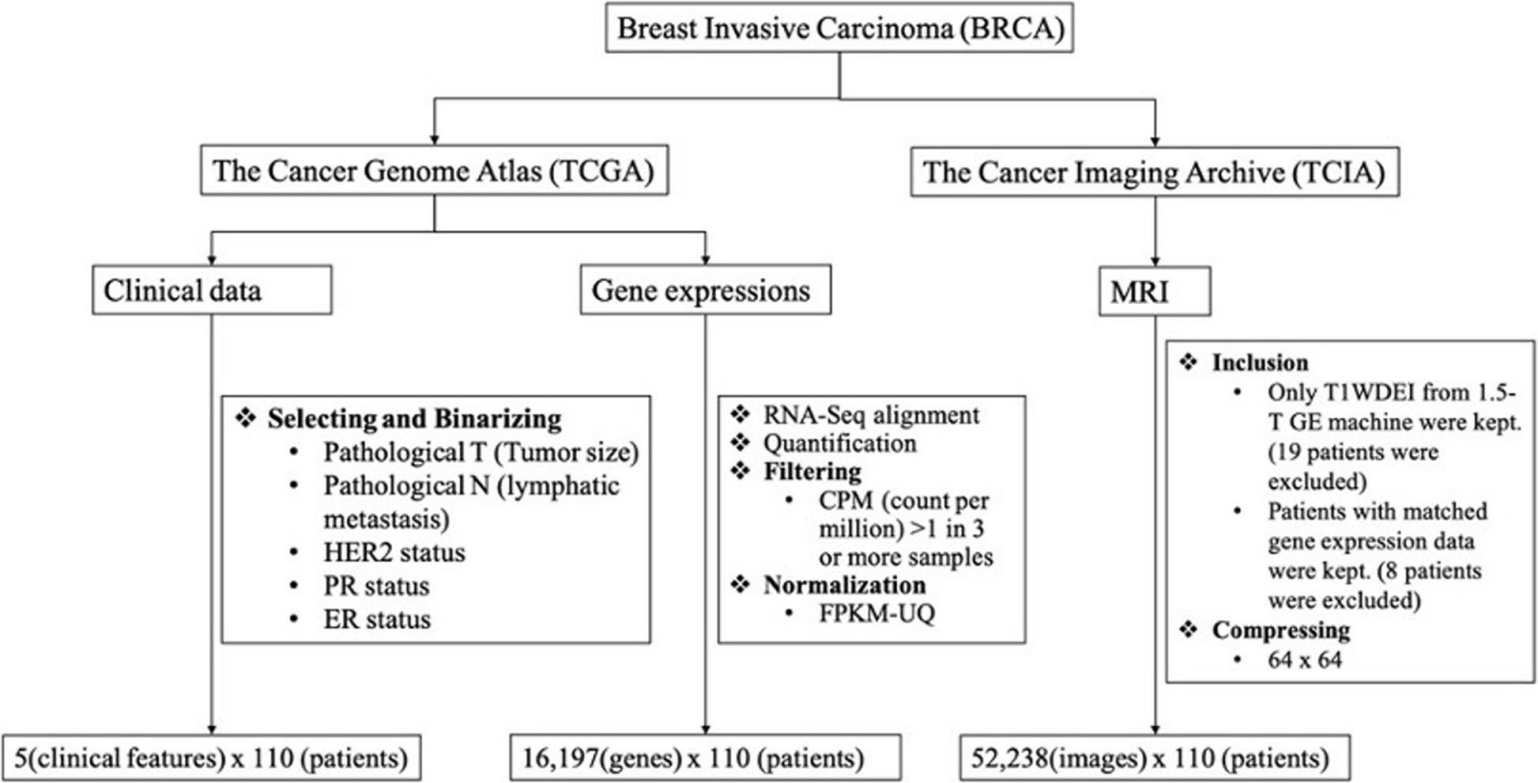
A flowchart showing the downloading and preprocessing procedures of the three data sources used in this study. Bold items were done by us, while RNA-Seq alignment and quantification were done by the TCGA database platform

Four datasets (MRI, CRFs, genomic data, and clinical data) of the same BC cohort were reused in this retrospective study. MRI of 137 patients were downloaded from The Cancer Image Archive (TCIA) [8]. This was all we can get from TCIA when this study was executed. Thirty-six CRFs for 87 of 137 patients were obtained from The Cancer Genome Atlas (TCGA) Breast Phenotype Research Group. Details of these 36 CRFs could be found in the original paper [4]. Genomic and clinical data were download from TCGA [9]. T1-weighted dynamic enhanced images (T1WDEI) from 1.5-T GE MRI machine were included in this study. Each patient has 2 to 8 post-contrast phases. We selected the specific phase that was used to obtain the CRFs so that the extracted DRFs are comparable. Twenty-seven cases that do not have T1WDEI from 1.5-T GE machine or do not have matched gene expression profiles were excluded. The exclusion criteria are shown in Fig. 1. The distribution of age and 5 clinical characteristics of the remained 110 patients are shown in Table 1.

**Table 1:** Participant Characteristics. Tumors with a size smaller than 2 cm were assigned to the T-negative group, while those with size larger than 2 cm were set to the T-positive group. Node metastasis was coded as N-positive/N-negative simply according to whether there were lymph nodes invasion or not. HER2, ER, and PR were binary already in the original data file we downloaded from TCGA

Parameter	Value
No. of participants	110
No. of women	110
Age	
Min	29
Max	82
Mean	53.76
Standard deviation	12.07
Pathological tumor size (T)	
No. of positive	70
No. of negative	40
Pathological lymphatic metastasis (N)	
No. of positive	54
No. of negative	56
ER	
No. of positive	86
No. of negative	24
PR	
No. of positive	77
No. of negative	33
HER2	
No. of positive	21
No. of negative	78
No. of NA^a^	11

^a^The 11 missing values for HER2 variable were not included in the clinical association analysis and the t-SNE map coloring

### Extraction of DRFs from BC MRI data

**Fig. 2:**
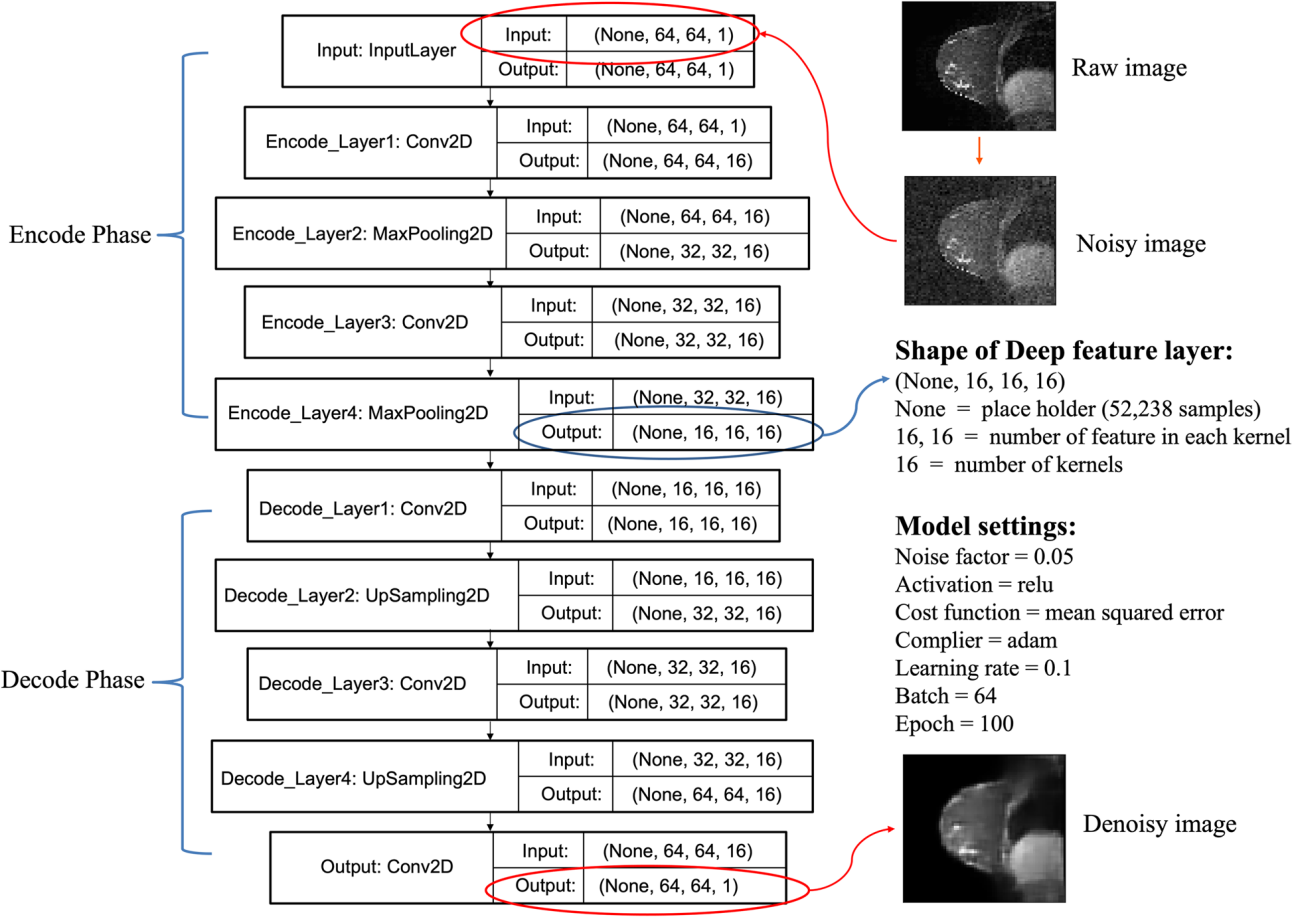
The DA model used in this study to extract deep radiomic features. There are two convolutional layers and two max-pooling layers in the encode phase, two convolutional layers, and two upsampling layers in the decode phase. For the (None, n, n, m), “None” is the batch size which we used to load the samples into the model, (n, n) in the middle indicates the number of features in each kernel. m is the number of kernels

A stacked convolutional denoising autoencoder (DA) [10] was build using Keras [11] (Fig. 2). DA is an unsupervised DL model that is widely used to extract intrinsic features from data[10]. We first scaled pixel values to the range of 0 to 1 in a min–max normalization way to improve computational efficiency. Then, images were interrupted with a normally distributed random noise as shown below:

$$noisy\_data\;\mathit=\mathit\;raw\_data\;\mathit+\mathit\;noise\_level\;\mathit\times\mathit\;random\mathit.normal\mathit\;\mathit(\mathit0\mathit,\mathit\;\mathit1\mathit,\mathit\;size\;\mathit(raw\_data\mathit)\mathit)$$

Here *noise_level* was 0.05, which means the noise we added into the data follows a normal distribution with mean equals to 0 and standard deviation equals to 0.05. Rectified Linear Unit (ReLU) and mean square error (MSE) were chosen as the activation function and the loss function. Adam was selected as the optimizer [12]. Learning rate, batch size, and epoch were set to 0.1, 64, and 100, respectively.

The dataset was split into train/test sets in a ratio of 80%:20%. After the model was well trained (loss was converged) and tested (no overfitting), we applied the model to the whole dataset and extracted the output of the last encode hidden layer, which encodes the information of the image to the most abstraction level, as our DRFs. There are 4,096 DRFs in this layer, which come from 16 kernels, and each kernel has a dimension of 16 $\times$ 16 (more details can be found in the source code).

### Normalization and visualization of DRFs

Quantile normalization was performed to make the DRFs comparable among samples [13]. Heatmap was employed to create the kernel-wise feature maps. Hierarchical clustering [14] and ﻿t-Distributed Stochastic Neighbor Embedding (t-SNE) [15] were used to cluster the normalized DRFs. Complete linkage function in the hierarchical clustering process and visual-guided criteria by analysis of the dendrogram were used to decide the number of clusters in the heatmap. T-SNE were done at both patient and image levels. Patient-level features were calculated as the mean of image-level features. After the patient-level t-SNE map was generated, colors were manually assigned to the visible clusters and the same colors were assigned to image-level t-SNE map to see if they have consistent patterns. We also colored the patient-level map with clinical characteristics to see if these clusters could be explained by existing clinical knowledge.

### Classification of clinical characteristics using radiomic features

We performed supervised classification analysis using radiomic features to predict the status of the 5 clinical characteristics of BC (tumor size (T), lymph node metastasis (N), estrogen receptor (ER), progesterone receptor (PR), and human epidermal growth factor receptor 2 (HER2) status) at image level. We did this separately for the learned DRFs and the downloaded traditional RFs. Since there are many predictors in the classification model, overfitting is likely to occur. We built a least absolute shrinkage and selection operator (LASSO) regression model using the R packages biglasso [16]. LASSO is a regularization technique that can be added into the fitting process to reduce the magnitude of coefficients so that overfitting could be avoided. The formula of the multiple linear regression is shown in the Eq. 1. The LASSO fitting is shown in the Eq. 2.

1 $$\begin{array}{cc}Y_i=\beta_0+\beta_1X_{i1}+\beta_2X_{i2}+\dots+\beta_pX_{ip}&i\mathit=\mathit1\mathit,\mathit\;\mathit2\mathit,\mathit\;\mathit\dots\mathit,\mathit\;N\end{array}$$

2 $$\widehat\beta^{lasso}=arg\;{min}_\beta\frac1N\sum\nolimits_{i=1}^N\left(-y_i\log(\beta_jx_i\right)-(1-y_i)\log(1-\beta_jx_i))+\lambda\sum\nolimits_{j=1}^p\vert\beta_j\vert$$

Here *X* is each of the radiomic features (RFs) (CRFs and DRFs). *Y* is a given clinical characteristic*. N* is the sample size. *p* is the number of the RFs in the feature vector. $\lambda$ is a hyperparameter used to control the level of penalty [16].

Models were trained on a randomly selected sample set with 70% of the total samples and performance was evaluated using a test set with the remaining 30% of the total samples. 100 $\lambda$ s were tried and the performances of the models with different $\lambda$ s in the training set were measured using a metric called area under the receiver operating characteristic curve (AUC_ROC) in a fivefold cross-validation way.

### Radiogenomic analysis

Radiogenomic analysis aims to evaluate the association between genomic profiles and DRFs. In this study, we focused on three levels of genomic features (GFs) extracted from the mRNA gene expression profiles of the 110 BC patients:

1. Gene expressions of 288 well-validated BC risk genes collected from previous studies [17, 18].
2. 6 commonly used BC gene signatures calculated using R package “genefu” [19]: Oncotype DX, EndoPredict, Prosigna (rorS), MammaPrint (GENE70), GENIUS and PIK3CA-GS.
3. 182 KEGG (Kyoto Encyclopedia of Genes and Genomes) pathway activity scores [20] calculated using the Single Sample Gene Set Enrichment Analysis (ssGSEA) function [21].

We performed association analyses between each RFs (DRFs and CRFs) and each of those GFs using a Linear Mixed Effect (LME) Model, which can model and analyze the complex and structured data with multi-levels [22]. In our case, multiple images can be obtained from each individual patient. We implemented the analysis using the R package nlme [23]. The formula of the LME model is as follows.

3 $$\begin{array}{cc}X_i=\beta_0+\beta_1G_i+\mu Z_i&i\mathit=\mathit1\mathit,\mathit\;\mathit2\mathit,\mathit\;\mathit\dots\mathit,\mathit\;N\end{array}$$

Here *X* is a given RFs, *G* is a given genomic feature and *i* is the *i*^th^ images. Since our DRFs are at image-level and each of the 110 patients has multiple images, the DRFs were not independent with each other. Therefore, to address the effect caused by the dependence of the DRFs, the term *μZ* was introduced to simulate the variations coming from the patient differences. Significant associations were selected based on the cut-off of adjusted *P*-values < 0.05 [24].

### Classification of gene signatures and TILs

In clinical, gene signatures (pik3cags, endo, gene70, genius, oncotypedx, rorS) and tumor-infiltrating lymphocytes (TILs) are very important for BC patients’ disease management and predicting the gene signature status and TILs status are believed to be much harder than predicting the basic clinic information (ER, PR, HER2, T, and N status). We calculated the 6 TILs (B cell, T cell CD4, T cell CD8, Neutrophil, Macrophage, Dendritic cell) using TIMER method [25, 26]. We then binarized the 6 gene signatures (pik3cags, endo, gene70, genius, oncotypedx, rorS) and 6 TILs (B cell, T cell CD4, T cell CD8, Neutrophil, Macrophage, Dendritic cell) using the first quantile as cut-off (top 25% were defined as positive, while the other 75% were defined as negative). We then built a DNN and a XGboost using R packages “nnet” and “xgboost”, respectively, to classify the binarized gene signatures/TILs based on the CRFs/DRFs. The performance of the models was evaluated in a training–testing way and was measured by AUC (more details about the hyperparameters could be found in the source code of DNN/XGboost models at https://github.com/qianliu1219/DA_BRCA_radiogenomics).

## Results

### Visualization of DRFs

**Fig. 3:**
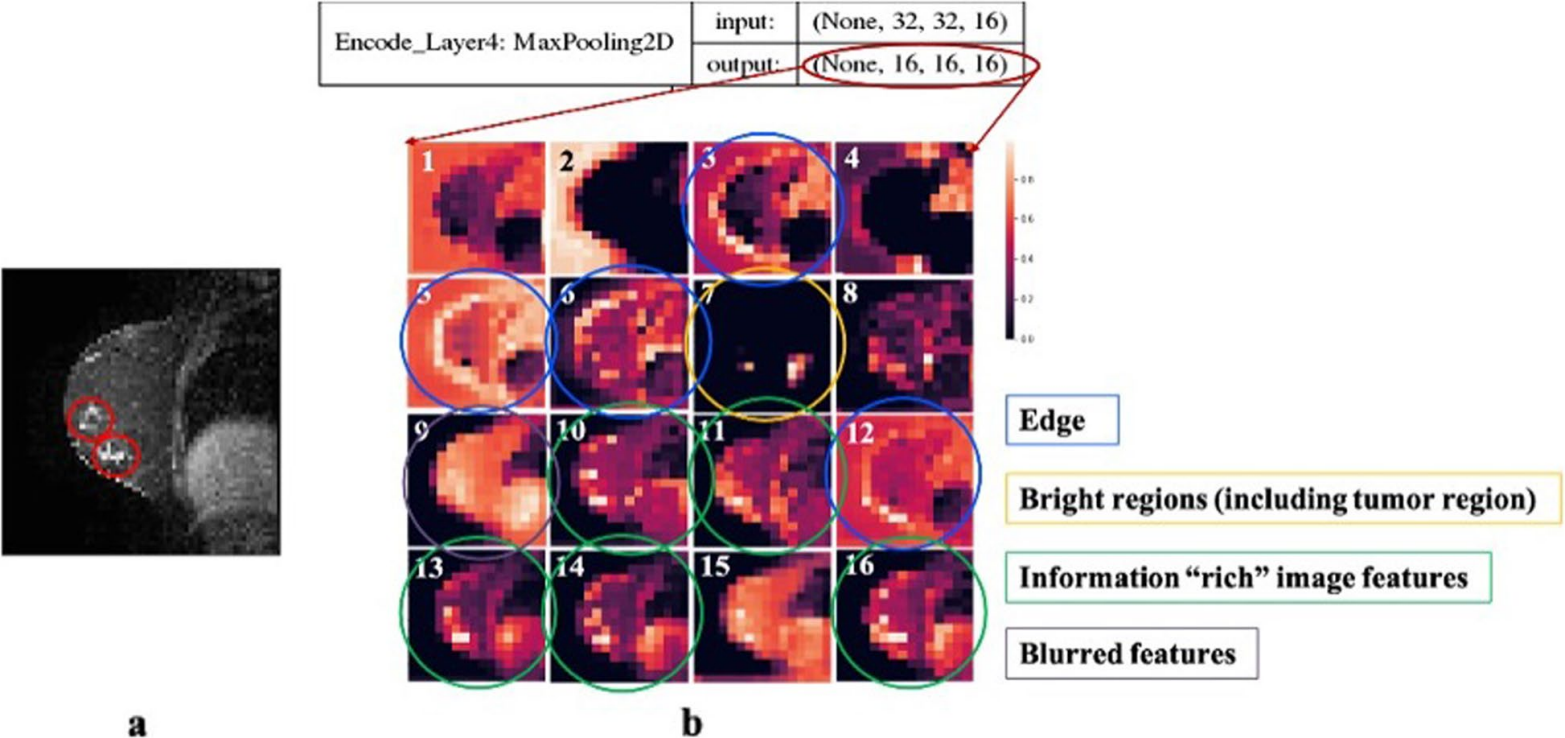
Visualization of kernel-level deep radiomic features (DRFs) by heatmaps. The values of the DRFs are presented by the magnitude of the colors in the heatmap. **a** A randomly selected raw image as an example. This MR image is a sagittal view of the body. Tumors are circled out. **b** The 16 kernel-wise heatmaps of the DRFs for the same image shown in a. Kernels #3, 5, 6, 12 are highlighting the edge of breast. #7 is showing the high value regions. Kernels #10, 11, 13, 14, 16 emphasize the tumor regions. #9 is smoothy and blur

To understand the potential biological and clinical meaning of the DRFs, we randomly selected several images (Fig. 3a is showing one of them) and visualized their 16 kernels using heatmaps (Fig. 3b). The 16 kernels are in high abstraction level and have learned different information from the original image. For example, kernels #3, 4, 5, 6, 12 highlight the edge of breast. Kernel #12 has almost absolutely reversed the signals in the diaphragm and tumor regions, but it puts a large weight on the bottom edge of the breast that is close to the tumor region, and there are some unclear patterns within the breast, chest, and lung regions. Kernel #5 highlights the edge of the breast without any bias. #7 only keeps the high pixel value regions including tumor and diaphragm. More interestingly, almost half of the heatmaps (kernels # 9, 10, 11, 13, 14, 15, 16) emphasize the tumor regions. Kernel # 9 and kernel #15 are smooth. Kernel #13 emphasizes the tumor regions while lowers the values of other regions inside and outside the breast, but it keeps reasonable values for the diaphragm region. Kernels #9, 14, 15, and 16 have the similar patterns as #13 but highlight the tumor regions in different magnitudes. Kernel #15 puts the similar values to the breast and diaphragm and slight weaker values to the chest. Kernels #14 and #16 are almost the same as #13 with a dimming in breast region.

### Unsupervised clustering analysis using the DRFs

**Fig. 4:**
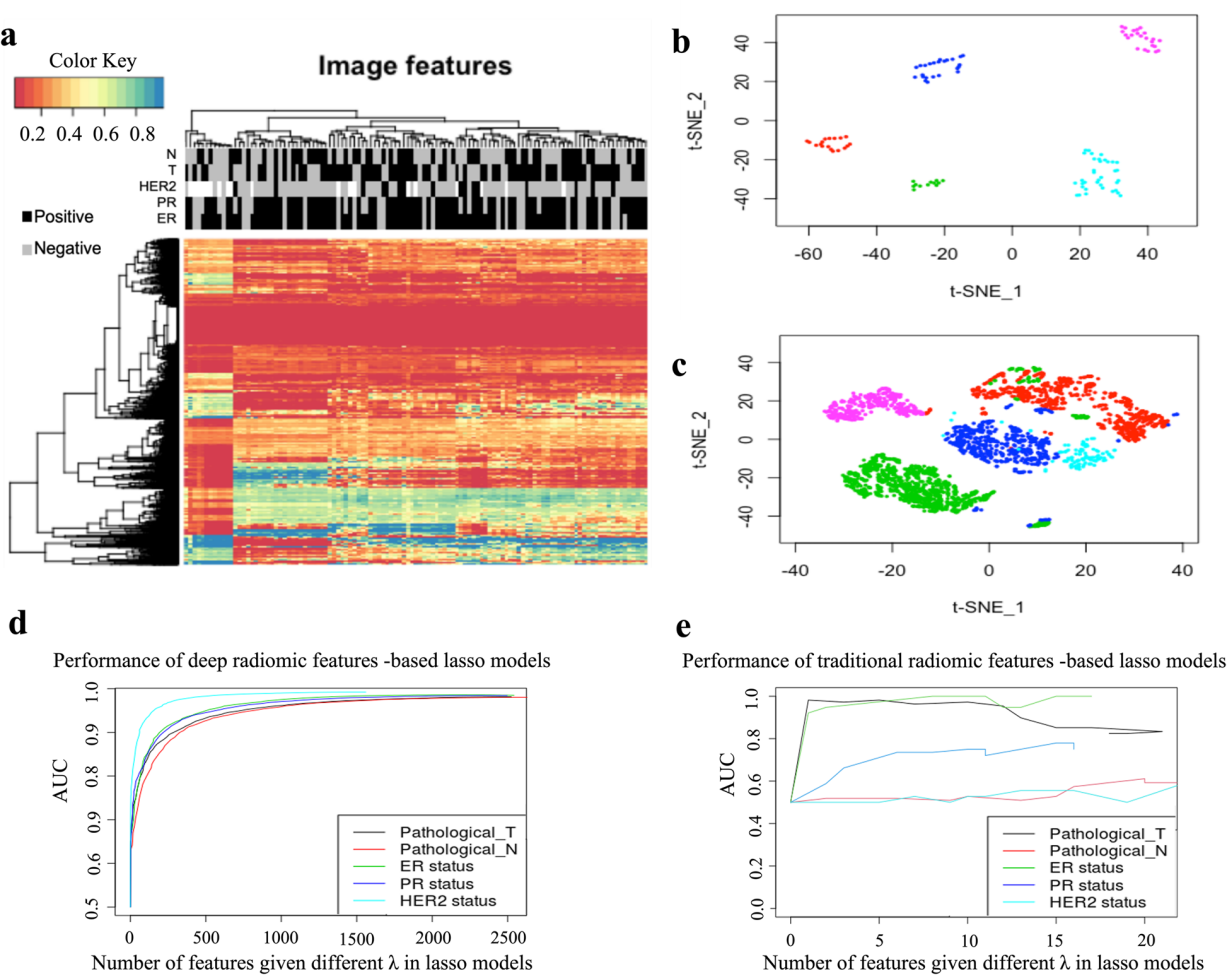
Unsupervised and supervised analysis results of the deep radiomic features (DRFs). **a** Unsupervised hierarchical clustering analysis of the DRFs. Columns are the 110 patients; rows are the 4,096 DRFs. Clinical information is shown in the sidebar. T refers to the tumor size. For breast tumors, bigger than 2 cm are T-positive. N refers to node status, which is positive when the tumor cell spreads into lymph nodes. ER, PR, HER2 refer to estrogen receptor status, progesterone receptor status, and human epidermal growth factor receptor 2 status. Patients seem to be clustered into 2 groups, but these two groups have no obvious clinical difference. **b** t-SNE visualizes the patient-level DRFs. Each dot is one patient. Different colors are marked in different patient-level clusters manually. **c** t-SNE visualizes the image-level DRFs. Each dot is one image. We first tracked the dots at image-level t-SNE map to patient-level, and then colored them using the same colors as what we used in coloring the patient-level t-SNE map. **d** The supervised LASSO model prediction performance of deep radiomic features under different $\lambda$ s. Different colors represent different clinical characteristics. The x-axis represents the number of deep radiomics features given different $\lambda$ in the LASSO models. **e** The supervised LASSO model prediction performance of traditional radiomic features under different $\lambda$ s. Different colors represent different clinical characteristics. The x-axis represents the number of traditional radiomic features given different $\lambda$ in the LASSO models. Please note that the feature number is not going up to the total number of features (4,096 or 36) because there were always a lot of features been regularized out under different $\lambda$ s. The y-axis represents the corresponding area under the curve (AUC) which is a metric used to assess the performance of the prediction. An AUC equals to 1 means a perfect prediction

The hierarchical clustering result of the normalized DRFs is shown in Fig. 4a. Patients were clustered into roughly two groups with unbalanced sizes. One has only 14 patients while the other has 96 patients. However, according to the sidebar labels, these two clusters do not enrich any of the five clinical characteristics (Fisher’s exact test, *P*-value > 0.05).

At the patient-level t-SNE map (Fig. 4b) and image-level t-SNE map (Fig. 4c), patients were clearly clustered into 5 groups. And the clustering patterns are consistent. However, we also did not observe the enrichment of any clinical characteristics in these five groups (Fisher’s exact test, *P*-value > 0.05). These 5 groups may have some other clinical differences (such as survival), but since all the 110 patients are still alive according to the latest follow-up information in TCGA, we do not have enough information to perform the survival analysis.

### Classification of clinical characteristics using DRFs

As the hyperparameter $\lambda$ increases, different numbers of RFs remain in the LASSO regression model to predict the clinical characteristics. Figure 4d shows the prediction performance of DRFs for the clinical characteristics using these LASSO models. Figure 4e shows the prediction performance of CRFs. Using LASSO models with proper $\lambda$, the DRFs performed very well (AUC could reach 0.90 or larger) in predicting the five clinical characteristics, which means the DRFs might be able to represent the combined information of these clinical characteristics. CRFs performed well in predicting pathological tumor size and ER status, however, their abilities of predicting other three clinical characteristics were weaker (AUC < 0.8).

### Association analysis between GFs and DRFs

**Fig. 5:**
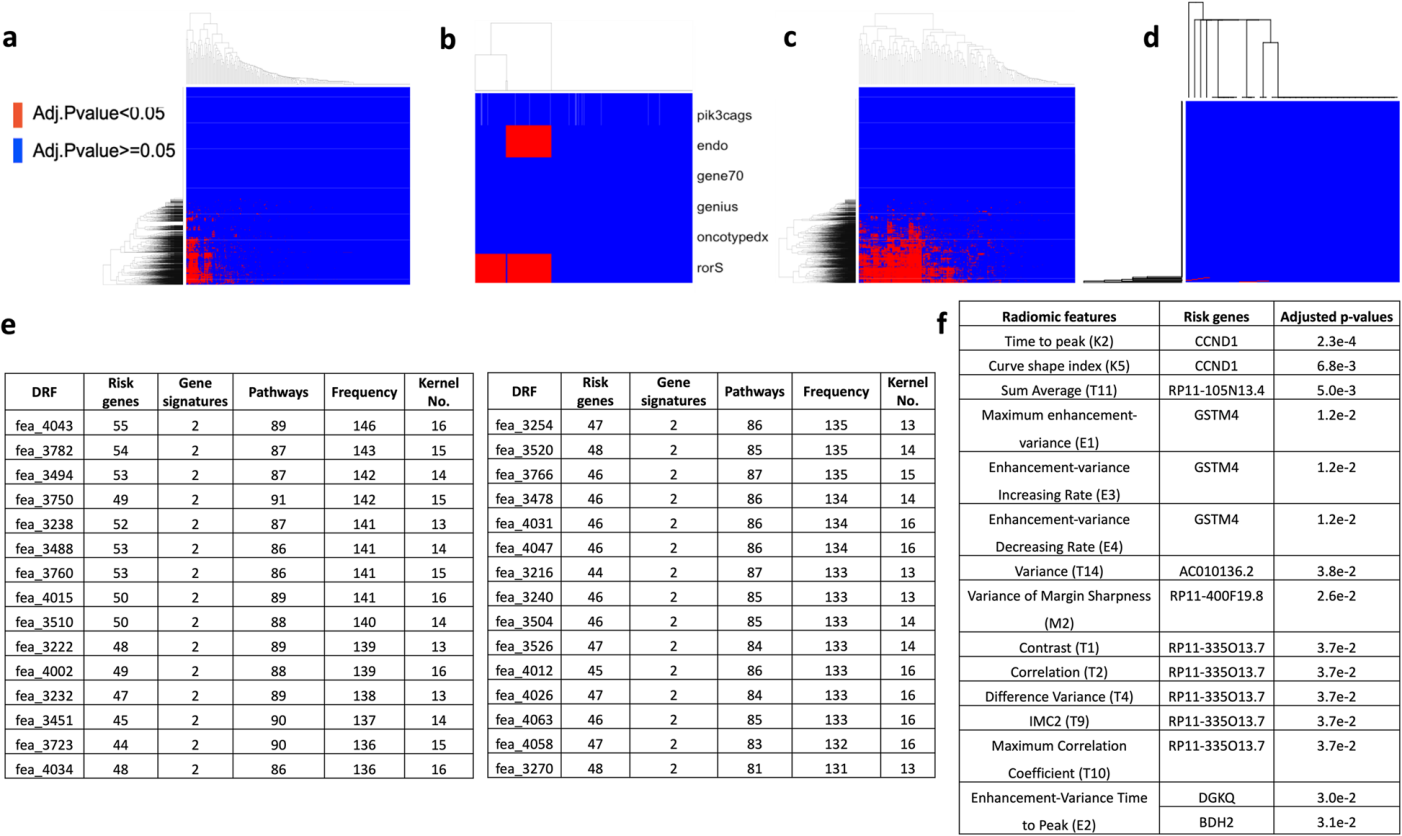
The results of radiogenomic association analyses between the radiomic features (RFs) and the genomic features (GFs). **a** the association results of 288 breast cancer risk genes and 4,096 deep radiomic features. **b** the association results of the 6 breast gene signatures with 4,096 deep radiomic features (DRFs). **c** the association results of the 182 KEGG pathway activity scores with 4,096 DRFs. **d** the association results of 288 breast cancer risk genes and 36 conventional radiomic features (CRFs). The association of gene signatures and KEGG pathway activity scores are not shown here because no significant associations have been identified. For **a**-**d**, the X-axis is the RFs. The Y-axis is the GFs. Red ones are significant ones. **e** the top 30 that are significantly associated with the GFs. Rows are the top 30 DRFs that have genomic significance (ranked by the frequency of the associated GFs). The first column is the ID of DRFs. The next three columns are the number of significant associations between the given DRF and the three sets of GFs, respectively. The fifth column is the accumulated number of significant associations that the given DRF has. The last column is the kernel where the given DRF is. **f** 14 CRFs are significantly associated with the 8 risk genes. Rows are the 14 CRFs that have genomic significance (ranked by the frequency of the associated GFs). The first column is the names of the CRFs. The second column is the risk genes. The third column is the adjusted *p*-values of the association analyses

After multiple testing correction, 1,774 out of the 4,096 DRFs were significantly associated with 213 of the 288 BC risk genes (Fig. 5a). On the contrary, only 14 CRFs were associated with 8 risk genes (Fig. 5d). The details of the 14 CRFs and the 8 risk genes are shown in the Fig. 5f. Two of the six gene signatures, EndoPredict and Prosigna (rorS) scores, are significantly associated with 848 and 1,395 DRFs (Fig. 5b), respectively. 1,739 of the 4,096 DRFs are significantly associated with 166 of the 182 KEGG pathways (Fig. 5c). CRFs have no associations with gene signatures and KEGG pathways activities. Totally there are 2,028 DRFs significantly associated with 381 (213 risk genes, 2 gene signatures and 166 biological pathways) GFs. The details of the top 30 DRFs that have the most significant association with the GFs are shown in Fig. 5e. Taking the top 1 DRFs in the first row as an example, the DRFs “fea_4043” is significantly associated with 55 BC risk genes, two gene signatures, and 89 KEGG pathways. Hence, there are 146 GFs significantly associated with the “fea_4043” located in kernel #16. Interestingly, these significant GFs are mainly associated with the DRFs from kernels #13 to #16.

We further calculated the number of significantly associated GFs with the kernel-level DRFs as shown in Table 2. The results in Table 2 are based on all 2,028 significant DRFs. As can be seen, these DRFs are mainly from kernel #12 to #16. Kernel #13 to #16 are considered as genetic information enriched kernels, because comparing with the first several kernels (e.g., #1 to #12), they all learned more abstract and representative information from their original images, which captured the tumor regions, put different weights to the surrounding tissues, and partially kept the signals for tissues far away from tumors. And among these top 5 DRF kernels (#12 to #16), #12 is special as it emphasizes the edge information, while kernels #13 to #16 more focus on the tumors and surrounding tissues.

**Table 2:** The frequency of the genomic associations with the kernel-level deep radiomic features (DRFs). Rows are each radiomic feature kernel. The first column is the kernel ID. The next three columns are the number of significant genomic features that are associated with the DRFs mapped in the given kernel. The last column is the accumulated number of significant genomic features that are associated with the DRFs within the given kernel. It should be noted that each kernel has 256 DRFs

Kernel	Risk genes	Gene signatures	KEGG Pathways	Frequency
13	3251	227	7848	11,326
14	3374	208	7423	11,005
15	3039	190	6776	10,005
16	3093	178	6650	9921
12	2510	203	6853	9566
11	2176	194	5934	8304
10	2045	185	5307	7537
9	1964	183	4706	6853
8	1852	163	3988	6003
7	1358	118	2752	4228
6	725	85	1601	2411
5	577	75	1399	2051
1	654	59	1158	1871
4	496	67	961	1524
2	358	56	890	1304
3	384	52	793	1229

The number of significantly associated DRFs for each GFs was also calculated. We reported the top 5 BC risk genes (RP11-57H14.3, FIBP, ATP6AP1L, OVOL1, RP11-400F19.8) that are significantly associated with the largest number of DRFs, the 2 significant gene signatures (EndoPredict, Prosigna), and the top 5 KEGG pathways (Fatty acid metabolism, Insulin signaling pathway, Phenylalanine metabolism, RNA degradation, Tyrosine metabolism) that are significantly associated with the largest number of DRFs (Table 3). As we can see, the DRFs associated with these top GFs are mainly from kernels #11 to #16.

**Table 3:** The genomic features that are associated with the largest number of deep radiomic features (DRFs). We only report the top five significant genomic features in each of the three categories (risk genes, gene signatures and KEGG pathways)

Genomic features	No. of significant DRFs	Top 5 radiomic feature kernels (ordered)
Risk genes		
RP11-57H14.3	1118	13, 14, 15, 12, 16
FIBP	1050	13, 14, 15, 10, 11
ATP6AP1L	1038	13, 14, 16, 10, 15
OVOL1	1019	13, 14, 10, 16, 11
RP11-400F19.8	1017	13, 14, 16, 12, 15
Gene signatures		
EndoPredict	848	13, 14, 12, 15, 11
Prosigna (rorS)	1395	13, 14, 12, 11, 10
KEGG pathways		
Fatty acid metabolism	1269	12, 10, 11, 13, 9
Insulin signaling pathway	1243	13, 12, 11, 14, 10
Phenylalanine metabolism	1217	12, 13, 10, 9, 11
RNA degradation	1211	12, 13, 11, 14, 15
Tyrosine metabolism	1205	12, 9, 10, 11, 13

### Classification of gene signatures and TILs

The performance (ROC and AUC) of the DNN and XGBoost classifiers could be found in Supplementary Fig. 1. Generally speaking, DRFs performed better than CRFs in predicting gene signatures (pik3cags, endo, gene70, genius, oncotypedx, rorS) and TILs (B cell, T cell CD4, T cell CD8, Neutrophil, Macrophage, Dendritic cell) using both DNN and XGBoost models.

## Discussion

We developed a DL model which could automatically extract DRFs from BC MRIs. These DRFs performed very well in predicting BC clinical characteristics, gene signatures and TILs, and have significant association with many GFs. We also visualized the extracted DRFs and made potential interpretations of the identified radiomic-genomic associations. 

Among the top 5 most significant BC risk genes in the association tests, the RP11-57H14.3 and the RP11-400F19.8 are processed transcript biotype. They do not code proteins and their biological functions are not clear [27, 28]. However, they were observed in several cancer related studies [29, 30]. FIBP, ATP6AP1L, and OVOL1 are protein-coding genes. The acidic fibroblast growth factor intracellular-binding protein [31] and the subunit ATPase [32] coded by FIBP and ATP6AP1L are related to cell metabolism and growth. The zinc finger protein [33] coded by OVOL gene could influence cell proliferation and malignant transformation by adjusting the MYC transcription, which is a well-known oncogene [34]. Please be noted that we only discussed the top 5 most significant risk genes. There are 213 such risk genes in total, gene ontology analysis or gene set enrichment analysis could be considered as potential future directions to explore them.

EndoPredict is a gene signature predicting the likelihood of distant recurrence in ER-positive and HER2-negative BC patients treated with adjuvant endocrine therapy [35]. Currently, the cost of a EndoPredict test is around 1,500USD [35]. Prosigna (rorS) score could be used to predict BC risk and it is calculated from PAM50, which is a set of proliferative genes [36]. A Prosigna test costs around 2,000 USD currently [37]. Progsigna and EndoPredict are all associated with kernel #11, 12, 13, 14 according to our radiogenomic association analyses, which indicates that they may capture the similar radiomic information and our DRFs may serve as surrogate to represent the information captured by these two commercialized gene signatures. Several metabolism pathways stand out in our radiogenomic association analysis, such as Fatty acid metabolism, Phenylalanine metabolism, and Tyrosine metabolism pathways. These pathways are all reported in BC studies [38], which could be used to support the rationality of our DRFs.

The proposed denoise autoencoder is unsupervised and can extract intrinsic features from the data itself without any external label information. This is because our primary goal is to perform radiogenomic analysis, that is, the association between genomic features and the radiomic features. In contrast, a supervised classification model may be forced to learn features that are only representing the label information. We want the proposed radiogenomic biomarkers can capture as much clinical/genomic information as possible. Thus, an unsupervised method (e.g. autoencoder) may be better for data-driven feature extraction than a supervised approach. Since using the extracted features to predict clinical characteristics is our secondary goal in the study, we also explored to use the unsupervised deep radiomic features from the denoise autoencoder combined with the LASSO. As a contrast, we built 5 pure supervised classification models using the famous EfficientNet (with and without pre-training on ImageNet) [39] for the 5 binarized clinical characteristics (ER, PR, HER2, T, N). For the supervised analysis, the dataset was split into train/test sets in a ratio of 80%:20%. Learning rate and epoch were set as 0.002 and 100. The losses were converged successfully for all trainings. The performances (AUCs) on test set is shown in Supplementary Table 1, which is comparable to the proposed unsupervised deep radiomic features combined with the LASSO method.

There are some limitations in the study. Firstly, although we discussed the potential biological meaning of the learned DRFs, the mechanisms of why those GFs are associated with certain DRF kernels are still unclear. Currently, there are no similar studies and interpretations to explain the biology meaning of the DRFs. Also, there has no good visualization tools for DRFs. The heatmap visualization we used for our kernel-wise DRFs were based on randomly selected samples, which may have limited generalization ability. Secondly, the sample size of the study is relatively small, so it is a bit hard to explain the clustering patterns of the patients identified by the tSNE. The patterns may be explainable in the future if the sample size for the breast cancer radiogenomic study is large enough. Thirdly, there is no other publicly available breast cancer radiogenomic dataset which can be used to conduct this kind of radiogenomic experiments. Thus, the extracted features cannot be replicated in another independent dataset at this stage. More validation needs to be done in the future when an independent cohort is available. Finally, lack of publicly available normal or benign breast MRI data also limited the findings of this study to be transferred to clinical applications. The comparison of the features extracted from the MRI of BC patients vs. features extracted from the normal or benign breast MRI should be performed to highlight the breast cancer-specific features.

## Conclusion

In summary, DL-based radiogenomics in BC was well-explored in this study. DRFs performed very well in predicting BC clinical characteristics and have significant association with many GFs. Potential biological interpretations were discussed to increase the transparency. The proposed method is fully automatic and could be transferred to any other image type as well as other diseases.

## Supplementary Information

**Additional file 1: Supplementary Table 1.** The performances (AUC) comparison of pure classifiers (EfficientNet with and without pre-training) and the unsupervised radiomic features combined with LASSO in clinical characteristics classifications. We tried to directly predict ER, PR, HER2, T, N status using the famous EfficientNet (with and without pre-training). The dataset was split into train/test sets in a ratio of 80%:20%. Learning rate and epoch were set as 0.002 and 100. The losses were converged successfully for all trainings. The pre-training was done using the ImageNet data. The implementation was executed using Python Keras package, which provides EfficientNet model structure and pre-trained parameters. **Supplementary Figure 1.** The performance of CRFs/DRFs in predicting BC gene signatures and TILs using DNN and XGboost classifiers. (see next page).

## Data Availability

The data used in the study can be accessible at TCGA platform (https://www.cancer.gov/tcga) and the TCIA platform (https://www.cancerimagingarchive.net/).

## Abbreviations

AUC_ROC: Area under the receiver operating characteristic curve
BC: Breast cancer
CRFs: Conventional radiomic features
DA: Denoising autoencoder
DL: Deep learning
DRFs: Deep radiomic features
ER: Estrogen receptor
GFs: Genomic features
HER2: Human epidermal growth factor receptor 2
LASSO: Least absolute shrinkage and selection operator
LME: Linear Mixed Effect
MRI: Magnetic resonance imaging
MSE: Mean square error
PR: Progesterone receptor
ReLU: Rectified Linear Unit
RFs: Radiomic features
TCIA: The Cancer Image Archive
TCGA: The Cancer Genome Atlas
t-SNE: ﻿T-Distributed Stochastic Neighbor Embedding
