# Supplementary material for: Radiogenomic association of deep MR imaging features with genomic profiles and clinical characteristics in breast cancer
Source: Biomark Res. 2023 Jan 24;11:9. doi: 10.1186/s40364-023-00455-y (PMC9872423; doi:10.1186/s40364-023-00455-y)
Supplement: Supplementary file 1 — Additional file 1: Supplementary Table 1. The performances (AUC) comparison of pure classifiers (EfficientNet with and without pre-training) and the unsupervised radiomic features combined with LASSO in clinical characteristics classifications. We tried to directly predict ER, PR, HER2, T, N status using the famous EfficientNet (with and without pre-training). The dataset was split into train/test sets in a ratio of 80%:20%. Learning rate and epoch were set as 0.002 and 100. The losses were converged successfully for all trainings. The pre-training was done using the ImageNet data. The implementation was executed using Python Keras package, which provides EfficientNet model structure and pre-trained parameters. Supplementary Figure 1. The performance of CRFs/DRFs in predicting BC gene signatures and TILs using DNN and XGboost classifiers. (see next page). [file 40364_2023_455_MOESM1_ESM.pdf]

Radiogenomic association of deep MR imaging features with genomic profiles and clinical characteristics in breast cancer

(Supplementary Material)

Qian Liu[1,2,3], Pingzhao Hu[1,2,4,5]

[1]Department of Biochemistry and Medical Genetics, University of Manitoba, 745 Bannatyne Avenue, Winnipeg, Manitoba, R3E 0J9, Canada

[2]Department of Computer Science, University of Manitoba, E2-445 EITC, Winnipeg, Manitoba, R3T 2N2, Canada

[3]Department of Statistics, University of Manitoba, 318 Machray Hall, Winnipeg, Manitoba, R3T 2N2, Canada

[4]CancerCare Manitoba Research Institute, 675 McDermot Avenue, Winnipeg, Manitoba, R3E 0V9, Canada

[5]Department of Biochemistry, Western University, London, Ontario, Canada

Correspondence:

Dr. Pingzhao Hu, Department of Biochemistry, Western University, Medical Sciences Building Rm. 342, London, Ontario, Canada, N6A 5C1.

Email: phu49@uwo.ca.

Supplementary Table 1. The performances (AUC) comparison of pure classifiers (EfficientNet with and without pre-training) and the unsupervised radiomic features combined with LASSO in clinical characteristics classifications. We tried to directly predict ER, PR, HER2, T, N status using the famous EfficientNet (with and without pre-training). The dataset was split into train/test sets in a ratio of 80%:20%. Learning rate and epoch were set as 0.002 and 100. The losses were converged successfully for all trainings. The pre-training was done using the ImageNet data. The implementation was executed using Python Keras package, which provides EfficientNet model structure and pre-trained parameters.

	Pathological_T	Pathological_N	ER status	PR status	HER2 status
EfficientNet[1]	0.96	0.97	0.98	0.97	0.95
EfficientNet (Pre-trained on ImageNet)	0.98	0.95	0.99	0.98	0.96
DA-DRF-LASSO[2]	0.97	0.97	0.99	0.98	0.99
CRF-LASSO[3]	0.97	0.61	0.99	0.79	0.59

[1]EfficientNet is a pure supervised deep learning approach;

[2]DA-DRF-LASSO is the LASSO classifier using the unsupervised radiomic features extracted from autoencoder;

[3]CRF-LASSO is the LASSO classifier using the conventional unsupervised radiomic features;

Supplementary Figure 1. The performance of CRFs/DRFs in predicting BC gene signatures and TILs using DNN and XGboost classifiers. (see next page)

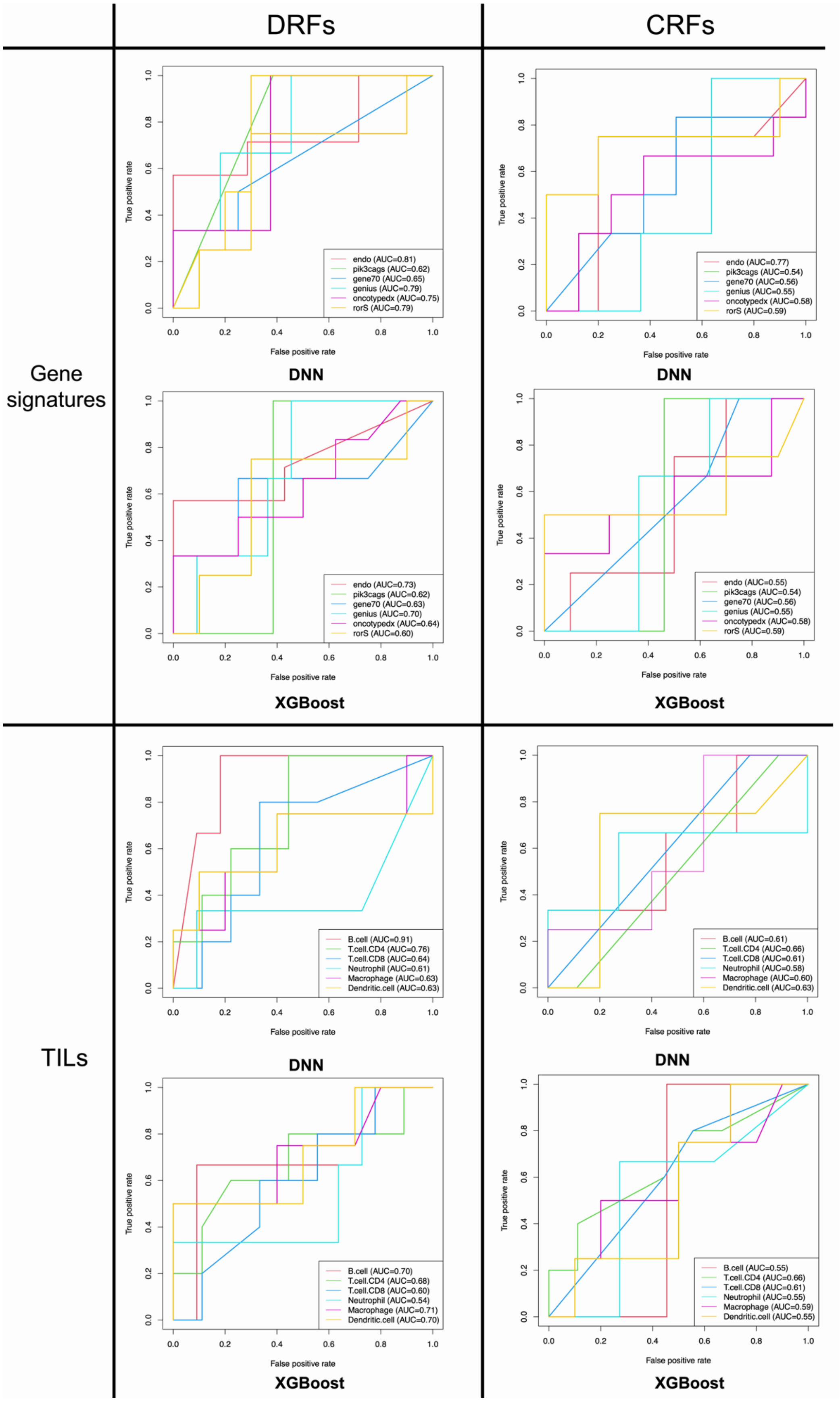
DRFs
CRFs
Gene signatures
TILs
True positive rate
False positive rate
endo (AUC=0.81)
pik3cags (AUC=0.62)
gene70 (AUC=0.65)
genius (AUC=0.79)
oncotypedx (AUC=0.75)
rorS (AUC=0.79)
DNN
endo (AUC=0.77)
pik3cags (AUC=0.54)
gene70 (AUC=0.56)
genius (AUC=0.55)
oncotypedx (AUC=0.58)
rorS (AUC=0.59)
DNN
endo (AUC=0.73)
pik3cags (AUC=0.62)
gene70 (AUC=0.63)
genius (AUC=0.70)
oncotypedx (AUC=0.64)
rorS (AUC=0.60)
XGBoost
endo (AUC=0.55)
pik3cags (AUC=0.54)
gene70 (AUC=0.56)
genius (AUC=0.55)
oncotypedx (AUC=0.58)
rorS (AUC=0.59)
XGBoost
B.cell (AUC=0.91)
T.cell.CD4 (AUC=0.76)
T.cell.CD8 (AUC=0.64)
Neutrophil (AUC=0.61)
Macrophage (AUC=0.63)
Dendritic.cell (AUC=0.63)
DNN
B.cell (AUC=0.61)
T.cell.CD4 (AUC=0.66)
T.cell.CD8 (AUC=0.61)
Neutrophil (AUC=0.58)
Macrophage (AUC=0.60)
Dendritic.cell (AUC=0.63)
DNN
B.cell (AUC=0.70)
T.cell.CD4 (AUC=0.68)
T.cell.CD8 (AUC=0.60)
Neutrophil (AUC=0.54)
Macrophage (AUC=0.71)
Dendritic.cell (AUC=0.70)
XGBoost
B.cell (AUC=0.55)
T.cell.CD4 (AUC=0.66)
T.cell.CD8 (AUC=0.61)
Neutrophil (AUC=0.55)
Macrophage (AUC=0.59)
Dendritic.cell (AUC=0.55)
XGBoost
